# Effect of time and storage temperature on canine and feline erythrocyte sedimentation rate

**DOI:** 10.1016/j.mex.2022.101934

**Published:** 2022-11-24

**Authors:** Eleonora Gori, Anna Pasquini, Daniela Diamanti, Carlo Carletti, Veronica Marchetti

**Affiliations:** a Department of Veterinary Sciences, University of Pisa, University of Pisa, Via Livornese snc, 56121, Pisa (PI), Italy; b DIESSE Diagnostica Senese Spa, Via Strada dei Laghi 35-39, Z.I., Casone, Ingresso 6 Monteriggioni, 53035, Siena (SI), Italy

**Keywords:** Red blood cells, Dog, Cat, Sedimentation, EDTA, Stability

## Abstract

We aimed to test the influence of storage temperature and time for canine and feline ESR. Forty dogs and 12 cats were included and randomly allocated in “room temperature” and “refrigerated” groups. Both groups had the T0 ESR measures few minutes after complete blood count. Afterwards, room temperature group had ESR measured at 2, 4, 6 and 8h after T0, whereas the “refrigerated” group had the blood sample stored at 4-6°C for 24 and then T24h ESR was measured. In each ESR measurement, [1] blood samples were put on a tube rocker waiting for ESR analysis; [2] before inserting the blood tube in the MINI-PET ESR instrument, samples were gently mixed again by complete inversion 10 times; (3) each mixed blood tube was inserted in the one of the four MINI-PET tubes position; (4) on the machine display, patient's species has to be chosen and the 14 minutes countdown started; (5) after the 14 minutes optical reading, the ESR result (mm/h) is displayed on the machine. ESR of canine samples at room temperature were significantly stable until T6, while feline samples remained stable at T8. After 24h at refrigerated temperature, both canine and feline samples were stable.

• MINI-PET is an ESR automatic continuous-loading instrument that can analyze up to four EDTA blood samples simultaneously using an optical system that measures the erythrocytes sedimentation level

• We aimed to test influence of storage temperature and time for canine and feline ESR

• At room temperature, dogs’ samples were stable within 6 hours from collection, and cats’ samples were stable until 8h. At refrigerated temperature, there was no difference in T0-T24 ESR in both canine and feline samples

## Graphical abstract

**Figure:**
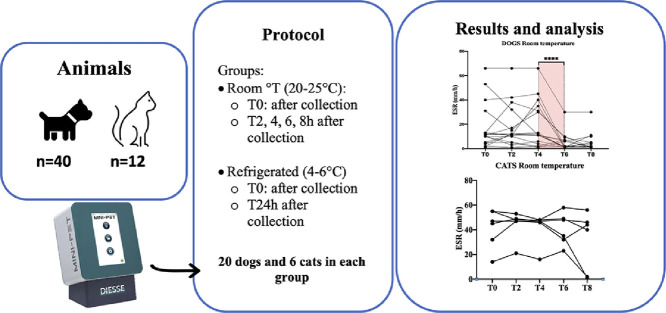

Specifications table

**Table:** 

Subject area:	
More specific subject area:	Clinical Pathology
Name of your method:	Erythrocyte Sedimentation Rate
Name and reference of original method:	C. Militello, A. Pasquini, A.A.M. Valentin, P. Simčič, G.D. Feo, G. Lubas, The Canine Erythrocyte Sedimentation Rate (ESR): Evaluation of a Point-of-Care Testing Device (MINIPET DIESSE), Vet Medicine Int. 2020 (2020) 3146845. https://doi.org/10.1155/2020/3146845.
Resource availability:	https://www.diessevet.it/it/mini-pet/

## Method details

The ESR was performed using the novel automated ESR device (MINI-PET; DIESSE, Diagnostica Senese S.p.A.) according to the manufacturer's instructions, also described in Militello et al. (2020). The MINI-PET is an ESR automatic, continuous-loading, instrument that can analyze up to four EDTA blood samples simultaneously, using an optical system that measures the erythrocytes sedimentation level (Militello et al., 2020).

**Fig. 1:**
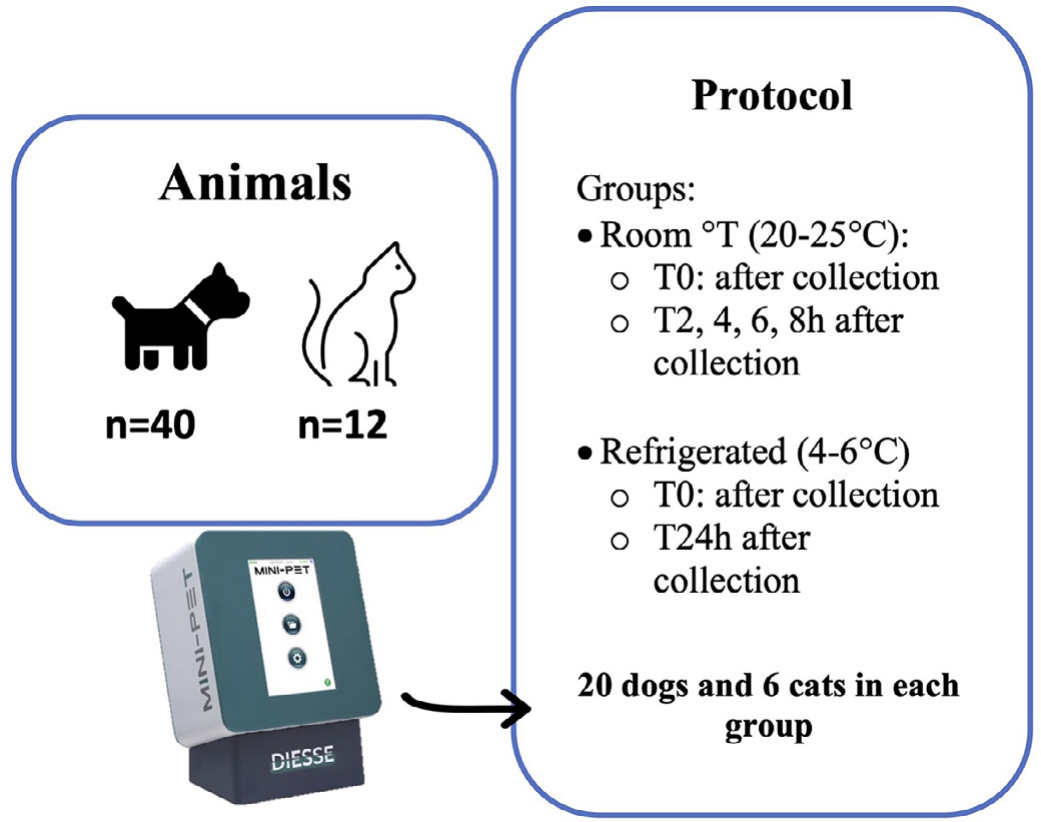
Enrollment and protocol procedure for ESR stability

Briefly, randomly selected feline and canine blood samples, collected in 1mL K3-EDTA tubes (Ø 12×56 mm; APTACA Spa, Canelli, AT, Italy) were used and randomly (https://www.random.org) assigned to “room temperature” or “refrigerated” group. As shown in Fig. 1, a total of 40 dogs and 12 cats were enrolled in this study. Both groups, “room temperature” and “refrigerated” group were composed by 20 dogs and 6 cats. Dogs allocated in room temperature group had their ESR measures at T0 (immediately after complete blood count execution and within 20 minutes from sampling), and after 2, 4, 6 and 8 hours after collection. Whereas dogs in the refrigerated group had their ESR measured at T0, then blood samples were refrigerated at 4-6°C for 24h, and the ESR was measured again 24 hours after collection after few minutes at room temperature.

In each ESR measurement, the procedure was the following:

- Blood samples were put on a tube rocker waiting for ESR analysis
- Before inserting the blood tube in the MINI-PET ESR machine, samples were gently mixed again by complete inversion 10 times
- Each mixed blood tube was inserted in the one of the four MINI-PET tubes position
- On the machine display, patient's species has to be chosen and the 14 minutes countdown started.
- After the 14 minutes optical reading, the ESR result (mm/h) is displayed on the machine.

If the “error” message was displayed, the K3-EDTA blood sample was removed from the machine, gently resuspended for 10 times and the test was newly attempted (Militello et al., 2020). If the “error” message could not be resolved, the sample was excluded from the study.

Data about each timepoint in the different study groups were recorded and analyzed to establish if there was any significant change in canine and feline ESR measurements. Statistical analysis was conducted using a commercial statistical software (SPSS v 22.0, IBM Corp.). The effect of time in the two temperature groups was tested using Friedman test and followed by Wilcoxon signed-rank test for nonparametric data to compare T0 ESR with subsequent ESR measurements in room temperature and refrigerated groups. A P-value < 0.05 was statistically significant.

## Method validation

**Fig. 2:**
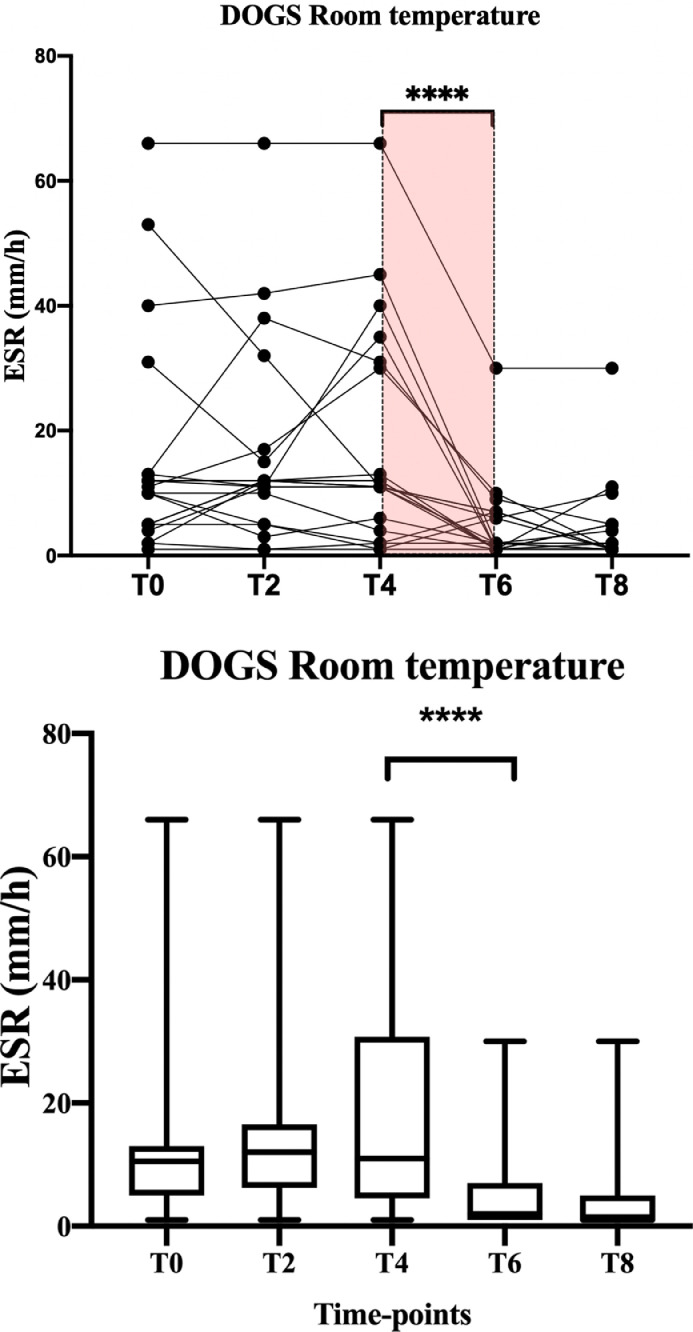
Graphical representation of canine ESR stability at room temperature, both with individual trend values (top line) and group trend (bottom line). The **** marked the statistically significant difference.

**Fig. 3:**
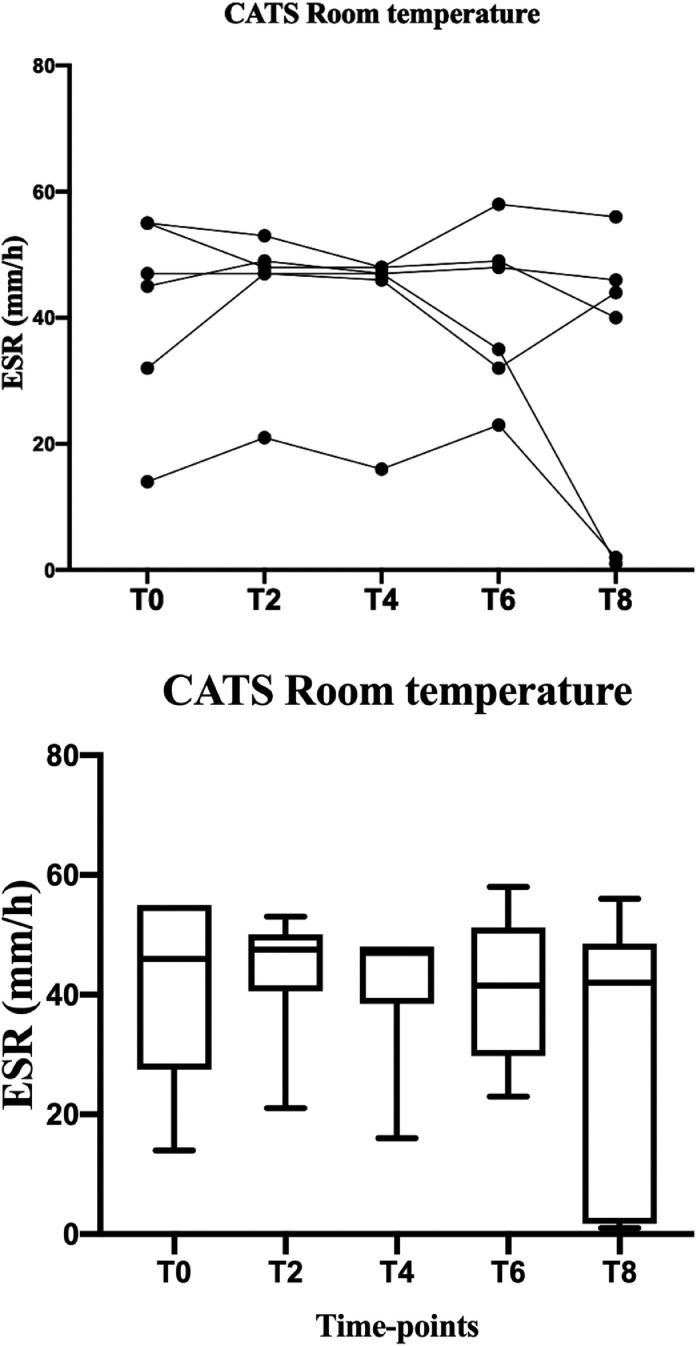
Graphical representation of feline ESR stability at room temperature, both with individual trend values (top line) and group trend (bottom line). The **** marked the statistically significant difference.

At room temperature the time affect the ESR of dogs (P<0.0001). As shown in Fig. 2 and 3, dogs’ ESR was stable within 6 hours from collection, afterwards ESR reduced abruptly (T4-T6 ESR P=0.0005). Meanwhile, time and temperature seem not to affect ESR in cats (P=0.2).

**Fig. 4:**
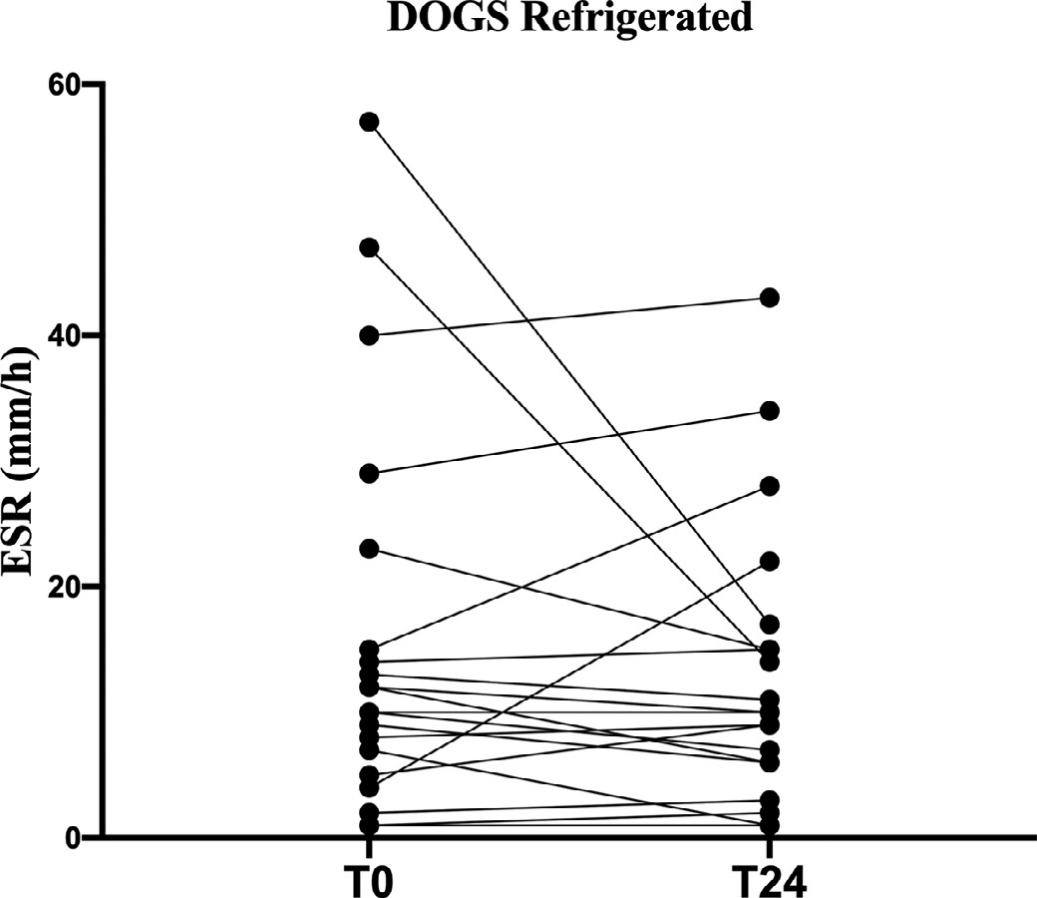
Fraphical representation of canine ESR stability at refrigerated temperature.

**Fig. 5:**
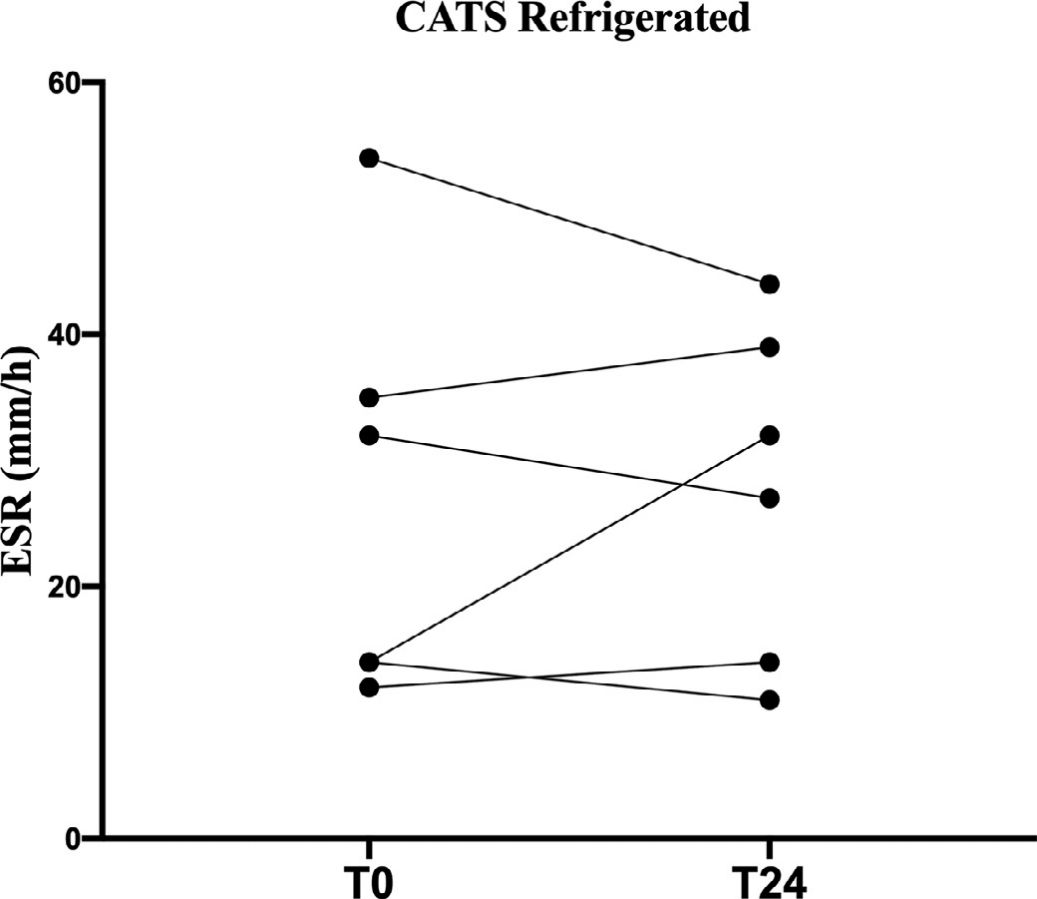
Graphical representation of feline ESR stability at refrigerated temperature.

In the “refrigerated” group, there was no significant difference in T0-T24 ESR in both canine and feline samples (p=0.51 and p=0.9, respectively; Fig. 4, Fig. 5).

## Ethics statements

This prospective study was performed on left-over blood samples of dogs and cats presented at the Veterinary Teaching Hospital of the University of Pisa between May 2022 and August 2022. Routinely, all owners presenting at the Veterinary Teaching Hospital must sign an informed consent for scientific use of their animals’ left-over blood samples. For this reason, a formal ethical approval was not requested.

## CRediT author statement

**Eleonora Gori:** Conceptualization, Methodology, Data curation, Writing- Original draft preparation. **Anna Pasquini:** Conceptualization, Methodology, Data curation, Writing- Reviewing and Editing. **Daniela Diamanti:** Conceptualization, Writing- Reviewing and Editing. **Carlo Carletti:** Conceptualization, Writing- Reviewing and Editing. **Veronica Marchetti:** Conceptualization, Writing- Reviewing and Editing, Supervision.

## Declaration of interests

The authors declare that they have no known competing financial interests or personal relationships that could have appeared to influence the work reported in this paper.

The authors declare the following financial interests/personal relationships which may be considered as potential competing interests:

DIESSE Diagnostica Senese Spa provided the MINI-PET ESR machine. Dr. Daniela Diamanti and Mr. Carlo Carletti are DIESSE Diagnostica Senese Spa employees. None of the other authors has any other financial or personal relationships that could inappropriately influence or bias the content of the paper.
